# Machine Learning and Deep Learning Techniques for Internet of Things Network Anomaly Detection—Current Research Trends

**DOI:** 10.3390/s24061968

**Published:** 2024-03-20

**Authors:** Saida Hafsa Rafique, Amira Abdallah, Nura Shifa Musa, Thangavel Murugan

**Affiliations:** 1 College of Information Technology, United Arab Emirates University, Abu Dhabi P.O. Box 15551, United Arab Emirates; 201350314@uaeu.ac.ae (S.H.R.); 700040171@uaeu.ac.ae (A.A.); nura.shifa@aau.ac.ae (N.S.M.); 2 College of Engineering, Al Ain University, Abu Dhabi P.O. Box 15551, United Arab Emirates; 3 Department of Information Systems and Security, College of Information Technology, United Arab Emirates University, Abu Dhabi P.O. Box 15551, United Arab Emirates

**Keywords:** anomaly, intrusion detection, Internet of Things, artificial intelligence, machine learning, deep learning

## Abstract

With its exponential growth, the Internet of Things (IoT) has produced unprecedented levels of connectivity and data. Anomaly detection is a security feature that identifies instances in which system behavior deviates from the expected norm, facilitating the prompt identification and resolution of anomalies. When AI and the IoT are combined, anomaly detection becomes more effective, enhancing the reliability, efficacy, and integrity of IoT systems. AI-based anomaly detection systems are capable of identifying a wide range of threats in IoT environments, including brute force, buffer overflow, injection, replay attacks, DDos attack, SQL injection, and back-door exploits. Intelligent Intrusion Detection Systems (IDSs) are imperative in IoT devices, which help detect anomalies or intrusions in a network, as the IoT is increasingly employed in several industries but possesses a large attack surface which presents more entry points for attackers. This study reviews the literature on anomaly detection in IoT infrastructure using machine learning and deep learning. This paper discusses the challenges in detecting intrusions and anomalies in IoT systems, highlighting the increasing number of attacks. It reviews recent work on machine learning and deep-learning anomaly detection schemes for IoT networks, summarizing the available literature. From this survey, it is concluded that further development of current systems is needed by using varied datasets, real-time testing, and making the systems scalable.

## 1. Introduction

The Internet of Things (IoT) has grown exponentially over the years, with its application spanning from healthcare to industrial devices. With its growth, it is providing an unprecedented level of connectivity like never before. The amount of data being produced has also increased exponentially as more devices are being connected. Sorting through this vast amount of data and organizing it in an ordered manner is a difficult task.

The IoT can be classified into either three-, four-, five-, or seven-layer architectures [1], while generally, the four-layer architecture is considered the essential component of the IoT [2]. These four layers are the Perception layer, Network layer, Middleware layer, and Application layer [2,3,4,5]. The Perception layer contains physical devices such as sensors and actuators that collect data for processing. The Network layer is the communication gateway for the Perception layer and the IoT system. The Middleware layer is where the collected data from the Perception layer are processed, stored, and managed. Finally, the Application layer contains the end-user applications that hold all of the processed data in meaningful values [4]. Other studies consider more layers to have an integral part in IoT architecture, such as the Security layer [6,7], Management layer [6], Business layer [1], and Environmental layer [8], which can also be considered as the Management layer.

IoT attacks are classified into four types: physical, encryption, network, and software-based attacks [9]. There has been a plethora of attacks in the IoT environment, namely buffer overflow attacks, brute-force attacks, DNS poisoning, injection attacks, replay attacks, DDoS attacks, SQL injection, back-door exploits, and more [10]. Additionally, research has been conducted revealing that the IoT can be used to facilitate violence between intimate partners who share a smart home [11]. Many attacks in the IoT can be prevented by using an anomaly detection mechanism, which can send an alert when any unusual behavior is detected. This can help in preventing attacks when they are attempted or indicating issues with system function that may result in downtime or failure. Table 1 highlights a summary of the attacks that occur in the IoT according to previous studies.

Anomaly detection is a security mechanism that distinguishes when a system’s behavior departs from the normal baseline [22,23]. It can be either host-based (HIDS) or network-based (NIDS) and is integral for IoT systems as it can detect variations in sensor readings, network abnormality, and so on [24,25]. Intrusion detection systems are categorized into three types: signature-based, anomaly-based, and stateful protocol [26,27]. Additionally, IDS methods can be implemented in three ways: supervised, unsupervised, or semi-supervised, which can be implemented through AI, statistical modeling, and so on [28]. To detect anomalies, the system first has to be trained on what behavior is normal in a given system and what the normal traffic pattern appears to be. Departure from this normality will be considered an anomaly [29,30]. Training the system will require a vast amount of complex IoT data with the usual network traffic pattern and considerable time to build a profile based on the IoT data [30]. Moreover, the traditional methods are not useful in detecting newer threats and need more time for updates [31], which can be mitigated with the use of artificial intelligence techniques such as machine learning (ML) and deep learning (DL) techniques. ML is a subfield of AI that comprises algorithms and models that help complete tasks through learning patterns and relationships rather than being explicitly programmed to do so. DL is a subset of ML that uses Artificial Neural Networks, which are more complex and can deal better with large amounts of complex data [32]. ML and DL techniques can use sophisticated analytical techniques to use the enormous and complex data of IoT systems cohesively to form a normal baseline for the network traffic of IoT devices [27,33]. This will result in improved accuracy, faster response times, cost-effectiveness, real-time detection, and more [31,34,35]. As a result, the ML and DL techniques can help detect when a system diverts from the baseline. ML and DL can detect anomalies by learning relationships and patterns from data, which can then be used to distinguish between normal and abnormal behavior. However, the differing factors between ML and DL techniques lie in their architecture and complexity, with DL being more complex as it deals with Neural Networks [32]. DL uses Neural Networks to learn a hierarchical representation of the data, which enables it to learn complex patterns [36]. ML and DL techniques combined with the IoT result in efficient anomaly detection that allows abnormalities to be found and fixed quickly. This strengthens the integrity, dependability, and effectiveness of IoT systems. This combination can also be used in any domain of the IoT, such as in healthcare, industrial settings, smart homes, and more [25,35,37,38].

To train ML and DL algorithms with IoT data, a dataset needs to be formed, which ideally should comprise real-time data of the IoT system. However, due to the complexity of IoT data, datasets are pre-formed by collecting the different types of traffic in IoT systems along with attack signatures. These datasets are used to train ML and DL algorithms and analyze the effectiveness of their various algorithms. Existing research mentions [39] that the datasets formed must simulate real-world settings and must be comprehensive and labeled. For the use of datasets in ML and DL techniques, the importance of feature extraction techniques, data cleaning, and conditioning routines are also emphasized. The accuracy of the dataset to real-world data will result in sound and reliable results from the AI algorithm detection. Commonly used datasets are the IoT-23, DS2OS, and Bot-IoT datasets, and more [24,39]. 

This paper examines the literature on investigating the technology and application domain of machine learning and deep learning-based anomaly detection in IoT infrastructure. This study focused on privacy and security issues related to the exchange and storing of patient data in intelligent health applications. The use of ML methods for Internet of Medical Things (IoMT) authentication and anomaly detection is also included in this study. The research also touches on the technology and use of ML approaches for anomaly detection in IoT networks. Furthermore, there is a focus on Hadoop-based big data processing frameworks and using ML techniques to identify anomalies in IoT networks. An additional discussion of the application of ML techniques in the domain of IoT anomaly detection, such as advanced ML techniques and intrusion detection systems, is also covered in this paper. Additionally, ML techniques for detecting distributed denial-of-service (DDoS) attacks in software-defined IoT networks are covered in the paper.

Considering DL-based anomaly detection, this paper investigates the technological and application fields of DL-based network intrusion detection systems, with an emphasis on IoT device security. It also focuses on the security of IoT devices by combining DL for intrusion detection with the IoT and IDSs. Using DL models for anomaly detection, this paper also examines studies that inspect the design of an intrusion detection system (IDS) for IoT networks. DL-based intrusion detection in IoT networks—more specifically, transport networks, clouds, and fog computing—is the application domain that is explored by most research in current times. This study also looks at DL-based cybersecurity for IoT systems in smart cities, with a particular emphasis on real-time anomaly detection in IoT data streams and time-series prediction.

This study presents a comprehensive review of the most recent work on ML and DL-based anomaly detection methods in the IoT. The study follows the following structure to achieve this: Survey Methodology, Machine Learning—IoT Network Anomaly Detection, Deep Learning—IoT Network Anomaly Detection, Research Summary, Research Gaps, Areas for Improvement, and Conclusions. In the Section 2, the methods used to retrieve papers for this study, and which years they were retrieved from, are explained. Following that, in the Section 3, the paper briefly explains the use of ML for anomaly detection. It then proceeds to present the recent literature on evaluating and studying different ML techniques for anomaly detection, which is further summarized in tabular format. In the Section 4, it discusses studies related to DL techniques in anomaly detection, which follows a similar structure to the previous section. The examples from the literature used to discuss anomaly detection in both the ML and DL sections are divided into papers on general anomaly detection and papers on attack-based anomaly detection for clarity. This study then summarizes some of the key points from the literature review in the Section 5. It then proceeds to explain the disadvantages and drawbacks of current research in the Section 6. Following this, in the Section 7, the study considers the future research that can be explored in this topic. At the end, in the Section 8, the main points of the study are highlighted, and the paper is concluded.

## 2. Survey Methodology

For this study, papers were collected from several publications that includes from most to least- IEEE, Elsevier, MDPI, Springer, ACM, Wiley, Hindawi, and others. Papers published in the years 2018–2024 were focused on to optimally analyze the recent studies conducted on the subject. Additionally, to maintain unbiased research in the study, both ML-based and DL-based papers were collected regarding anomaly detection in the IoT. Each study was analyzed according to the domain in which it was carried out, the problem statement it addressed, the process of the experiments conducted (input–process–output), the datasets used, the advantages of the frameworks proposed, and the results obtained.

**Figure 1:**
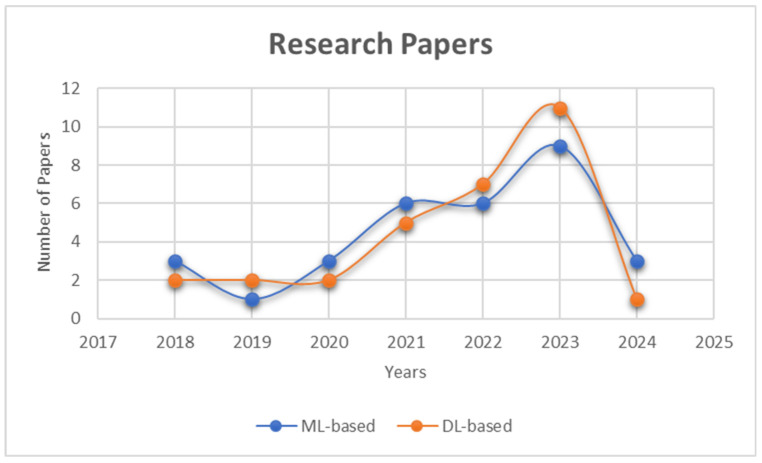
Number of papers considered for this study from 2018 to 2024.

For this study, a total of 60 papers were considered for the literature review of both ML-based and DL-based techniques. Figure 1 shows the publication years of the papers collected for this study in graph form. For both ML-based and DL-based papers, there has been an increase in publications over the years, with more DL-based papers being published in 2023.

## 3. Machine Learning—IoT Network Anomaly Detection

**Figure 2:**
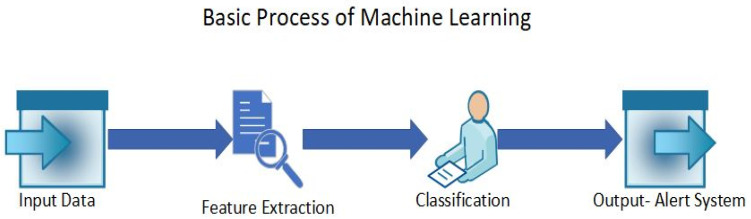
The basic process of a machine learning algorithm trained with IoT data to detect an anomaly.

Machine learning-based (ML-based) anomaly detection is highly researched and is a valuable technique for identifying anomalies in IoT systems [40]. There are four methods of learning in ML, which are supervised learning, unsupervised learning, semi-supervised learning, and reinforcement learning [41]. In supervised learning, the system is trained on labeled datasets and the system explicitly identifies the anomalies. However, unsupervised learning depends on the structure of the data, as it uses unlabeled data, hence the anomaly is detected according to the structure of the data [42]. ML is said to be effective in detecting anomalies and threats in real time [43]. The use of ML in the IoT also provides scalability, real-time decision making, predictive maintenance, resource optimization, automation, and more [44,45,46]. The different algorithms in ML can be used to optimize the detection of anomalies and implement it in various industries in real time. Figure 2 shows the basic process of ML algorithms, with the input data being labeled or unlabeled IoT data and the output being an alert system that mentions whether the data are anomalous or normal.

The study of ML-based anomaly detection is categorized firstly into papers that discuss anomaly detection and IDSs, which includes anomaly detection and attacks, and secondly into attacks that occur in IoT networks. Attacks in the IoT are anomalies in the context of anomaly detection as the attacks require the system to portray unusual behavior for it to be successful. The traffic could be anomalous through malicious payload, behavioral anomalies, unusual network traffic, and such. Hence, a comprehensive study was conducted into different aspects of anomaly detection with the use of ML. The summary of all the studies and their results for anomaly detection using ML is presented in Table 2, and attack-based anomaly detection is presented in Table 3. 

### 3.1. Anomaly Detection

The study in [47] uses machine learning approaches to investigate the identification of anomalies in the IoT context. It makes use of two datasets for time-series data and databases such as the NSL-KDD dataset for non-time-series data. Numerous classification methods, such as K-Nearest Neighbors, Multilayer Perceptron, Decision Trees, Linear Discriminant Analysis, Logistic Regression, and Naïve Bayes, are compared in the study. The findings demonstrate that, for non-time-series data, Decision Trees and Linear Discriminant Analysis produce consistent outcomes with 80% accuracy. When dealing with time-series data that has underlying trends, Neural Networks equipped with memory gates perform better than other techniques. A comprehensive picture of anomaly detection in IoT environments was provided by the study’s evaluation of time-series and non-time-series data. However, the small number of datasets utilized could be a drawback, as it could affect how broadly applicable the findings are. 

Using an ML-based approach to detect and prevent attacks and anomalies in IoT sensors, the study in [48] attempted to address cybersecurity concerns in IoT infrastructure. To show how well-suited basic models like Decision Tree (DT) or Random Forest (RF) models are for anomaly detection, the study evaluated the effectiveness of several ML models in terms of accurately predicting attacks and abnormalities on IoT systems using open-source datasets from Kaggle. The results of the study showed that Random Forest and Artificial Neural Network (ANN) techniques outperform Decision Trees in terms of testing and training accuracy with an accuracy rate of 99.4%, while Support Vector Machine and Logistic Regression methods are less effective. While the paper proposed models that can detect attacks with high accuracy, it also notes that the datasets only contain certain types of attacks and anomalies and hence they may not be scalable in real IoT environments.

To prevent system failure, the research in [49] proposed ways to address the problem of detecting attacks and abnormalities in IoT systems. It suggested a novel approach to a feature-transformation-based classifier for the classification and imputation of missing data values and evaluated its performance on real datasets. The classifiers it examined were Naïve Bayes (NB), Decision Tree (DT), Support Vector Machine (SVM), and Random Forest (RF) models with the DS2OS dataset. The suggested approach replaces missing values in a dataset using state-of-the-art imputation technology, and then it uses a feature transformation strategy to lower the dataset’s dimensionality and improve classification performance. The findings demonstrated that the proposed strategy beat baseline approaches in terms of performance metrics including F1 score, accuracy, precision, and recall. The accuracy rate of detection for the Random Tree and Decision Tree classifiers were 99.43% and 99.44% respectively. The study proved that the proposed method managed to retrieve missing values from the data, and identified anomalies. It also points out that more datasets are needed to carry out further research in order to form a sophisticated framework.

Regarding datasets, one study [50] explored the use of ML techniques to identify anomalies in IoT networks. They tested a system that used IoT device network traffic data to extract important information and train models like the K-Nearest Neighbors (KNN), Decision Tree (DT), and Random Forest (RF) models. Real-time network traffic anomalies were identified using these models, and the system classified traffic as regular or abnormal. The IoT-23 dataset was used in the experimental setup, and the RF algorithm performed the best in identifying abnormalities, producing an F1 score of 0.999 and an accuracy of 99.9% with a weighted average precision of 1.00. The study also considered security indicators like true positive, false positive, true negative, and false negative rates. The study showcases accurate detection of anomalies in the IoT, while also suggesting further research with more varied datasets.

Furthermore, in the domain of industrial sensors, the study in [32] analyzed recent ML-based anomaly detection schemes for IoT networks, identified their drawbacks, and proposed a novel scheme that combines supervised and unsupervised ML algorithms which is said to perform better than existing techniques in terms of recall, precision, and F1 score. It classified various ML models such as Random Forest and Decision Tree models and highlighted that these ML models present low false positive rates, which hinders their accuracy and resilience. For increasing the accuracy of anomaly detection, the study suggests testing the models with more data, which could be challenging to obtain, due to the privacy of data that could be solved with a central server to deal with all traffic. Moreover, the study also shows that the N-BaIoT dataset is a comprehensive IoT dataset, covering most dimensions and anomalous attacks.

Another study [39] was conducted focusing on datasets and finding abnormal traffic patterns in IoT networks, which can lead to issues with privacy and security. The UNSW-NB15 and DAD datasets were used by these authors to create intelligent security solutions. They used five shallow learning techniques along with ML to identify anomalies in traffic, namely the Naïve Bayes (NB), Logistic Regression (LR), AdaBoost (AB), Random Forest (RF), and Support Vector Machine (SVM) techniques. The study evaluated the effectiveness of various ML approaches and validated the DAD dataset. AB and RF performed the best, with a mean accuracy of 0.9998 each. Validating the DAD dataset and creating intelligent defenses for IoT networks are benefits of this research. However, some ML approaches and dataset restrictions present potential downsides of the study.

Considering the Wi-Fi network domain, the study in [51] focused on indoor positioning challenges, signal attenuation, and the need for precise anomaly detection. It assessed the effectiveness of different ML models for anomaly detection, suggested a method based on ensemble learning for better accuracy, and provided an experimental analysis of the proposed approach. The proposed system uses a self-organizing map (SOM) to classify RSS data from Wi-Fi routers into normal and problematic categories. The aberrant data are then exposed to a method of ensemble learning, including the elliptic envelope method, Random Forests (RFs), and Decision Trees (DTs). The system outputs a normal or abnormal categorization of the RSS data. The results showed that the suggested ensemble learning strategy performed better in terms of recall, precision, and F1 score than alternative algorithms like Decision Trees and Random Forests. When combining the stacking ensemble method with the RF method, an accuracy of 98% was achieved compared to the RF alone, which gave 94.7% accuracy. This portrays that ensemble learning improves the accuracy of anomaly detection; nonetheless, to achieve high accuracy, a large dataset is also required.

IoT devices are susceptible to security breaches because of their constrained feature and resource sets. One study [52] sought to find irregularities in IoT devices. It suggested a Hadoop-based architecture for IoT anomaly detection that makes use of ML classifiers, evaluated its efficacy, and contrasted it with other solutions already in place. The system trained the K-Nearest Neighbors (KNN), Support Vector Machine (SVM), and Naïve Bayes (NB) classifiers with pre-processed IoT device data and found anomalies in real-time data streams. ToN-IoT and BoT-IoT were the two datasets employed in the experimental setup to assess the efficacy of the framework. The findings demonstrated that the suggested framework outperformed other intrusion detection systems and outperformed existing methods in terms of F1 score, accuracy, precision, and recall. The accuracy detection of the proposed approach with the BoT-IoT dataset was 99% and with the ToN-IoT dataset was 90%. The study’s proposal achieved accuracy with low false positive rates; however, more research is recommended using larger datasets and more complex ML algorithms.

Finding a balance between accuracy and efficiency in real-time security operations for IoT applications was the goal of another study [53]. It evaluated memory consumption, execution time, and anomaly detection accuracy for ML-enabled models, which were the Logistic Regression (LR), Decision Tree (DT), Random Forest (RF), and Gradient-Boosting Machine (GBM) models. To decrease the execution time, memory consumption, and detection error rate, the study developed a Pareto-optimal collection of models. GBM and RF outperformed other algorithms with 99.99% accuracy, fast execution speed, and low memory consumption, according to the results. The models in the study showcased high accuracy with low detection error, execution time, and memory usage. To further improve this research, a larger, more complex, and more varied dataset is required to better detect anomalies in real IoT systems.

For applications in smart homes, the research in [54] developed an ML-based framework for estimating energy use in smart homes using previous consumption data. The system used data from smart devices and sensors, along with environmental data, to forecast and detect anomalies. ML methods like Artificial Neural Networks (ANNs), Prophet, LightGBM, and Vector Autoregression (VAR) were employed. The system generated energy consumption forecasts and searched for abnormalities in power usage patterns. Real-world data from a smart home were used to train and test the ML algorithms for anomaly identification and predictions. The results showed that the ML models performed well in identifying anomalies and estimating energy usage, with the Prophet and LightGBM models outperforming the VAR model for point anomaly identification. LightGBM achieved the most accuracy, with a mean absolute error (MAE) of 0.282046. The study stated that this technology can help with smart home automation and power system maintenance. To be able to do so, the models need to be further trained with larger and more varied datasets.

The research in [55] addresses the issue of label noise in IoT intrusion detection, which has an impact on the efficiency of ML algorithms. To identify noisy data, the authors provide a framework that combines uncertainty sampling and active learning with Decision Tree classification. This method works well for label noise detection as it can reduce the amount of noisy data identified by up to 98%. The study’s benefits include a significant detectable proportion of noisy samples with a low number of evaluated samples, explainable AI premises, and comprehension for non-expert users. The study’s emphasis on binary classification, however, might not apply to other classification issues, and its generalizability might be constrained by the particular datasets employed.

Similarly using the IoT-23 dataset, another study [56] explored the use of ML methods to enhance security in IoT systems. It focused on the application of the Gradient-Boosting and Extreme Gradient-Boosting (XGBoost) algorithms in identifying anomalous traffic. The studied system used IoT device network traffic data as the input, extracted features after pre-processing, and trained XGBoost to identify unusual traffic. The system’s output then predicted if network traffic was abnormal. The experimental setup involved obtaining network traffic data from IoT devices with both normal and abnormal traffic from the IoT-23 dataset. The ML algorithm was trained and assessed using XGBoost, and the results showed that XGBoost performed better than other algorithms like Support Vector Machines (SVMs) and Deep Convolutional Neural Networks (DCNNs) in terms of accuracy, precision, recall, and F1 score. XGBoost achieved accuracy levels as high as 99.98% during the execution time. Employing XGBoost will improve IoT security as it will accurately detect anomalies; however, further testing is needed with larger datasets and real-world situations.

A new framework for automated ML-based intrusion and anomaly detection in IoT networks was developed by another study [57]. Concerning criteria like server count, error rate, and network protocol, its goal was to predict network invasions. To function, the suggested system needed to first analyze network traffic from IoT devices, preprocess the data, and transfer it to an Edge server for storage, and then it used an ML model, which was the Bagged Tree (BT) model, to classify the data as either normal traffic, anomalies, or attacks. Network traffic was labeled by the system as routine, attack, or anomaly. This model achieved a 99.79% classification accuracy, which was higher than previous models. The model’s performance indicators included the ability to distinguish between 22 different categories of anomalous behavior and types of attacks, reducing the likelihood of attacks and speeding up reaction times. Despite its high accuracy rate, the model had poor performance during the testing period.

In the paper of [58], an anonymized network traffic approach to network anomaly identification in IoT networks is presented. The study proposed an ML model that can handle activities including feature extraction, network monitoring, anonymization, the training of models, and device identification as a suggested solution. The proposed model used a combination of the K-Nearest Neighbors (KNN), Logistic Regression (LR), and Multilayer Perceptron (MLP) methods. The findings indicate that while anonymization preserved an accuracy of 99.5% (achieved by KNN) in network anomaly detection, it decreased the capability for IoT device identification. The study includes limitations, such as its use of only one dataset and the need for more evaluation, but the proposed approach provides advantages relating to privacy and adheres to privacy standards.

Introducing a new dataset, the research in [59] focused on anomaly detection in IoT environments, addressing limitations of sensing devices’ power, bandwidth, and memory. It introduced a novel CoAP-IoT dataset for anomaly detection, validated these data using supervised learning techniques, and proposed an ML-based IDS that overcame previous solutions. The system used pre-processed data from the CoAP-IoT dataset, extracted relevant features, and trained a classifier to classify traffic as either normal or abnormal. The experimental setup of the study [15] used the CoAP-IoT dataset, and various supervised learning methods were used to train the system. The results showed that the Naïve Bayes classifier performed poorly, while the Support Vector Machine, Logistic Regression, and Decision Tree-based classifiers were comparable with a mean accuracy of 0.9.

The research in [60] proposes a system for intrusion detection based on quick protocol processing and feature grouping to address the difficulty of ensuring IoT device security. Four ML models were employed to measure performance: Decision Tree (DT), Random Forest (RF), K-Nearest Neighbors (KNN), and Extreme Gradient-Boosting (XGB). Three public IoT datasets were used to evaluate these methods, which were BoT-IoT, MedBIoT, and MQTT-IoT-IDS2020. The proposed system produced classification with high F1 scores with an F1 score of more than 0.99 for all datasets. Its effective and lightweight approach offers interpreted characteristics to expose the mechanisms of malicious attacks, making it appropriate for IoT devices with constrained processing and storage capacity. Nevertheless, this technique might not work with different IoT protocols or circumstances.

### 3.2. Attack-Based Anomaly Detection

The difficulties of detecting distributed denial-of-service (DDoS) assaults in actual networks using ML approaches are covered in the study in [61], with particular attention paid to data loss and incorrect classification of valid traffic. To train and evaluate ML algorithms, these researchers employed Packet Capture (PCAP) information from the Information Security Centre of Excellence (ISCX) in Canada. The goal of the study was to create classifiers for testing against DDos attack scenarios by choosing seven well-known classifiers, namely the QDA, SVM, KNN, Naïve Bayes, Decision Tree, and Random Forest classifiers, then identifying attributes defining network traffic patterns. To identify and categorize DDoS attacks in a real network testbed, they used the Data Plane Auxiliary Engine (DPAE) in nmeta2, an SDN-based traffic categorization architecture. The DPAE performed better with less processing time and a higher number of predictions. However, from the ML models, the SVM had a high accuracy of prediction, with a 0.93 mean accuracy score. The study showed the use of SDN for centralized network control, which can be used to detect anomalous traffic. However, it also highlighted some challenges regarding legitimate traffic being misclassified and the processing time of the models, which can cause delays. 

The study in [62] focused on identifying DDoS attacks that compromise IoT infrastructure by coming from consumer IoT devices that are not secure. Using data from regular and DDoS attack traffic collected from an end-user IoT device network, the researchers generated a dataset for training and testing ML algorithms. They experimented with five distinct ML classifiers: Neural Networks (NNs), Decision Trees, Random Forests, Support Vector Machines with linear kernels, and the K-Nearest Neighbors algorithm. The test set accuracy of all five classifiers was greater than 0.99, indicating the efficacy of combining ML methods and IoT-specific data for DDoS detection. With excellent recall, accuracy, F1 scores, precision, and total precision, the Neural Network classifier produced the greatest results.

The difficulty of defending against distributed denial-of-service (DDoS) attacks in Industrial IoT (IIoT) scenarios is covered in the paper in [63]. It proposes a fog/edge computing and federated learning collaborative defense strategy. The UNSW NB15 dataset was used by the proposed FLEAM procedure for both training and assessment. A global optimized model was collaboratively trained following the proposed protocol using distributed datasets from several defenders. The outcomes included a 47% increase in accuracy, a 72% reduction in mitigation reaction time, and an accuracy equivalent to that of centralized training.

A hybrid approach was suggested in another study [64] that focused on the security of IoT networks, a challenge faced by manufacturers who often fail to follow security requirements. A two-stage hybrid approach was proposed using advanced ML algorithms, namely Support Vector Machines, ensemble classifiers, and Decision Trees, along with a genetic algorithm, to detect intrusions in IoT networks. The system used pre-processed network traffic data extracted using a genetic algorithm, classified the traffic as dangerous or normal, and output an alert for intrusions. The system was trained and tested using a multi-class NSL-KDD dataset, achieving an accuracy of 99.8% using 10-fold cross-validation. The results showed that the ensemble classifier, Decision Trees, and SVMs, among other classifiers, had overall classification accuracy values of 99.8%, 99.5%, and 99.2%, respectively. However, the study mentions that the ensemble classifier was proven to yield the best results.

In IoT networks, the problem of identifying and preventing malicious Bot-IoT traffic is covered in [65]. In this paper, a new feature selection measure approach called CorrAUC is evaluated using a Bot-IoT dataset. To increase the accuracy of detecting malicious communication, this algorithm filters features. With an average accuracy of over 96%, the approach demonstrated exceptional specificity, sensitivity, accuracy, and precision in identifying Bot-IoT attacks. However, although the study offers a thorough framework for handling security issues in IoT networks, it is not scalable or evaluated using a particular dataset.

The identification of malicious intrusions in network traffic before they cause harm to an organization was the subject of another study [66]. It looked at the connection between five different categorization algorithms’ sensitivity and the quantity of packets observed. The researchers put forth a novel technique for identifying assaults when network traffic is just getting started. The suggested system classifies network traffic as harmful or benign based on pre-processed data. Using the CSE-CIC-IDS2018 dataset, the experimental setting evaluated the performance of five classification techniques: Random Forest (RF), Decision Tree (DT), K-Nearest Neighbors (KNN), Gaussian Naïve Bayes (GNB), and SVM. The results showed that the suggested method, which only included the first 10 packets of every flow, achieved excellent accuracy and efficiency in identifying malicious assaults with RF classification, achieving a high F1 score of 89.5% and precision of 99.38% in detecting attacks. The authors also conducted a sensitivity study to examine how various hyperparameters affected the effectiveness of the suggested strategy.

With an emphasis on security in IoT networks, the study in [67] explored the application of ML methods to categorize IoT malware. For learning techniques, it made use of datasets IoT-23, NetML-2020, and LITNET-2020. Algorithms including Random Forests (RFs), K-Nearest Neighbors (K-NN), and Artificial Neural Networks (ANNs) were used in the suggested solution. The RF algorithm achieved the highest accuracy in detecting attacks, with an accuracy score of 96%. However, handling huge datasets and algorithmic restrictions are potential drawbacks, as they can become computationally heavy in IoT systems.

The study in [68] employed network intrusion detection and ML models to identify and stop IoT-Botnet attacks. The training and evaluation datasets were derived from CICIoT2023. The Gaussian Naïve Bayes (GNB), Random Forest (RF), K-Nearest Neighbors (KNN), and Decision Tree (DT) classifiers were all used in the suggested solution. At 99.17% accuracy, the DT classifier proved to be the most precise. The benefits of this approach include its large dataset, in-depth analysis, and strong defense against Botnet attacks that are always changing. On the other hand, the detection of anomalies unique to IoT and computational complexity present possible challenges.

To detect DDoS attacks with the use of ML models, one study [42] focused on detecting DDoS attacks in software-defined IoT networks, a challenge exacerbated by the unpredictable nature of these networks and the increasing complexity of DDoS attacks. The research proposed an ML-based model for identifying DDoS incidents in IoT networks, comparing its performance to current solutions. The proposed system uses ML methods such as Naïve Bayes (NB), Decision Trees (DTs), and SVMs to categorize network traffic data as malicious or benign, with three primary parts: classification, feature extraction, and data pre-processing. The system filters and cleans raw network traffic data, extracts features, and uses the ML methods to determine if a communication is malicious or normal. The system’s output thus indicates whether the communication is malicious or legitimate. The results showed that the proposed framework maintained a low false negative rate while outperforming current approaches in terms of detection and false positive rate, with the DTs achieving the highest accuracy rate of 98.1%. ML-based anomaly detection can be used in various domains; however, a challenge remains with the datasets used, as they are not formed with diverse traffic and varying application devices. Moreover, the data used are not real-time, which remains a challenge [42].

To identify cyberattacks on Industrial IoT (IIoT) networks, the study in [69] proposes a hybrid ML technique. This methodology employs a mixed range of ML techniques to create a hybrid ML (HML) model to discriminate between legitimate and malicious traffic. Ten ML classifiers were combined to make the HML, including KNN, GB, LR, RF, ET AB, LDA, and CART. The efficacy of the technique was assessed using the sophisticated open-source DS2OS dataset. The accuracy rate achieved with the model was 99.8%, with an F1 score of 99%. The model achieved a high rate of accuracy and F1 measure in classifying malware, but it could be computationally heavy when implemented.

The study in [70] investigated the security issues with IoT devices and how ML algorithms can identify and avert security breaches. Using a laptop, an Alexa device, and a HomePod that were all linked to a router, the authors carried out a real-world experiment in which they attacked the network with an ARP poisoning attack. They suggested classifying and identifying unwanted traffic on the Xiaomi Redmi Note 9S device using the Decision Tree (J48) method. The outcomes demonstrated that the algorithm was successful in maintaining privacy and gaining security improvements. However, the paper fails to go into extensive detail about accuracy rates or performance indicators.

In [71], comparative research of different ML models highlights the difficulties in identifying vulnerabilities used by cyberattacks as it addresses the problem of detecting intrusions in IoT networks. It makes use of the IoT network intrusion dataset as well as the IoT-23 dataset. Using these datasets, the study evaluates five ML classifiers, namely Random Forest (RF), Decision Tree (DT), Naïve Bayes (NB), Multilayer Perceptron (MLP), and K-Nearest Neighbors (KNN), to detect intrusions. The best-performing algorithms, RF and DT, exhibit accuracy scores of 99.9% each for both the IoT-20 and IoT-23 datasets. The paper provides insights into the performance of ML models for detecting network intrusions and highlights the need for more research with larger datasets to improve the accuracy of these models.

To improve cybersecurity in IoT systems, the study in [72] addresses how to use the IoTID20 dataset to identify and stop denial-of-service (DoS) assaults. To keep an eye out for DoS abnormalities in network data, the authors suggested implementing ML classification methods in an IDS. Feature selection based on correlation-based feature selection (CFS) and the use of genetic algorithms (GAs) were used to train K-Nearest Neighbors (KNN), Decision Tree (DT), Random Forest (RF), and Support Vector Machine (SVM) classifiers. According to the study, training RF and DT with 100% accuracy using GA-selected features produced the greatest results across all assessment criteria. Unfortunately, the study also has some shortcomings, like its inadequate consideration of DL methods for IDSs in IoT systems and its lack of attention to scalability.

The study in [73] examines how vulnerable IoT security systems are to hostile attacks, with a particular emphasis on ML-based intrusion detection, malware, and device identification systems. The Smart Home Testbed dataset, UNSW-NB15 dataset, CIFAR-10 dataset, Kitsune dataset, Bot-IoT dataset, and NSL-KDD dataset are all used in the study. It categorizes attack-generating strategies and defense mechanisms and assesses how well various methods work with ML classifiers, including Naïve Bayes (NB), Random Forests (RFs), Support Vector Machines (SVMs), Decision Trees (DTs), and the J48 Decision Tree. Subjected to adversarial attacks, the Random Forest classifier showed impressive resilience, with the accuracy declining by only 21%. Although this study provides insights into cutting-edge adversarial approaches, it might have drawbacks such as restricted balanced IoT datasets.

The study in [74] addresses the growing issue of malware assaults and suggests efficient methods for their detection and classification with the use of the UNSW-NB15 dataset. The suggested strategy optimizes the performance of ML models such as LR, KNN, DT, ET, RF, and MLP through the application of Deep Convolutional Network techniques and image processing. This study’s technique outperformed earlier techniques with an accuracy rating of 99.98% when using the ET classifier. However, there were several drawbacks, such as its requirement of multiple datasets, the possibility of overfitting, and the failure to address zero-day attacks, which pose serious cybersecurity risks.

**Table 2:** Summary of the ML-based literature for anomaly detection in the IoT environment.

Ref.	Problem Addressed	Dataset	Proposed Solution	Results Obtained	Advantages	Disadvantages	Year
[47]	Anomaly detection in the IoT using ML	Time-series data, NSL-KDD	Compared several ML classifiers, such as KNN and DTs	DTs and Linear Discriminant Analysis achieved 80% accuracy with non-time-series data	Both time-series data and non-time-series data used	Small dataset size	2018
[48]	Detecting anomalies and attacks in IoT networks	Dataset from Kaggle	Compared different ML models in predicting attacks and anomalies on IoT systems	RF and ANN outperformed DT with 99.4% accuracy	Model better and faster than other techniques	Dataset limitations and computational complexity	2019
[49]	Identifying attacks and anomalies in IoT systems	DS2OS	Proposed a new method for missing data values, and evaluated its effectiveness on real datasets	The accuracy rate was 99.43% with RT and 99.4% with DT in detecting anomalies	Managed missing data values, reduced dataset dimensionality	Needs testing with more datasets	2020
[50]	Identifying anomalies in the IoT using ML	IoT-23	Investigated ML models, compared algorithms, and assessed their performance using metrics	RF outperformed others with an accuracy rate of 99.9%	Accurately identified anomalies in network traffic	Needs testing with more datasets other than IoT-23	2020
[32]	ML-based anomaly detection	-	Proposed combining supervised and unsupervised algorithms	Showcased different ML models, datasets, and applications	A broad overview of all related topics	In-depth research is needed	2021
[39]	ML-based anomaly detection IoT networks	DAD and UNSW-NB15	Used five shallow ML models (NB, LR, AB, RF, and SVM) with the DAD dataset	RF and AB achieved a mean accuracy of 0.9998	Dataset validated in detecting anomalies with ML	Needs varied datasets and testing with different ML models	2021
[51]	Anomaly detection in indoor Wi-Fi and IoT devices	UCI Wi-Fi indoor localization dataset	Evaluated ML models and proposed ensemble learning for improved accuracy	Ensemble learning strategy with RF achieved an accuracy of 98%	Precise anomaly detection method for indoor IoT devices	Needs more training data and testing with more ML algorithms	2021
[52]	Using ML-based models to detect anomalies in the IoT	ToN-IoT and BoT-IoT	Proposed a Hadoop-based framework using KNN, SVM, and NB ML classifiers	Accuracy was 90% with ToN-IoT and 99% with BoT-IoT	High accuracy and low false positive rates	Needs testing with larger datasets and more complex ML algorithms	2021
[53]	ML-based anomaly detection in the IoT	DS2OS	Assessed memory usage, execution time, and detection accuracy for LR, DT, RF, and GBM	GBM and RF achieved 99.99% accuracy, outperforming others	Minimizes detection error rates and execution time	Requires extensive training data	2022
[54]	ML-based techniques to identify power consumption anomalies	Private data	Employed ML-based techniques—VAR, Prophet, and LightGBM	LightGBM had the best accuracy with an MAE of 0.282046	Can help in smart home automation and power system maintenance	Needs more testing with large data	2022
[55]	Detecting label noise in IDSs with ML models	-	Proposed new framework using Decision Trees and active learning to detect label noise	Reduced noise by 98%	An explainable AI approach that detects a high % of noise	Only achieves binary classification, and uses a limited dataset	2022
[56]	Identifying anomalous activity in IoT systems with ML	IoT-23	Evaluated Gradient-Boosting and Extreme Gradient-Boosting (XGBoost) techniques using the IoT-23 dataset	XGBoost had a high accuracy rate of 99.98%	XGBoost can increase IoT system security	Needs more testing on larger and real-world datasets	2022
[57]	Network intrusions and cyberattacks in the IoT with ML	KDDcup99	Used BT ML model to test anomalies and compared it to other models (KNN, NN, SVM, etc.)	Model accuracy was 99.79%	Distinguished between 22 different anomalous behaviors	Performed poorly during testing	2023
[58]	Detecting anomalies and maintaining user privacy with ML	Real-life traffic data from IoT device networks	Proposed an ML model using KNN, LR, and MLP that identifies IoT devices and detects anomalies	Accuracy of 99.5% was achieved with KNN while keeping the device anonymous	Ensures the privacy of users while accurately detecting anomalies	Needs more testing on datasets and in varied networks	2023
[59]	Anomaly detection in the IoT with ML	CoAP-IoT	Introduced a new CoAP-IoT dataset and validated it using supervised learning	RF, SVM, and DT performed best, with a mean accuracy of 0.9	Created a new dataset and validated it	Needs more testing using various datasets and real-world IoT systems	2023
[60]	ML-based IDSs in the IoT	Bot-IoT, MedBIoT, and MQTT-IoT-IDS2020	Introduced a lightweight framework by testing it with DT, RF, KNN, and XGB ML models	Achieved high classification accuracy with an F1 score of 0.99 across all datasets	Lightweight and efficient, which is suitable for IoT applications	The ML model used does not apply to all IoT sensors	2023

**Table 3:** Summary of the ML-based literature for attack anomaly detection in the IoT environment.

Ref.	Problem Addressed	Dataset	Proposed Solution	Results Obtained	Advantages	Disadvantages	Year
[61]	DDoS attacks in the IoT	PCAP data	Used ML classifiers QDA, SVM, KNN, NV, DT, and RF	DPAE outperformed other models	SDN-based categorizer	Legit traffic misclassified and delays in detection	2018
[62]	Detecting DDoS attacks in the IoT	Regular DDoS attack traffic data	Five ML classifiers tested with datasets—NN, DT, RF, SVM, KNN	Classifiers achieved an accuracy of more than 0.99. NN achieved the best overall	Accuracy in detecting DDoS attacks	No real-world dataset used	2018
[63]	DDoS in IIoT scenarios	UNSW NB15	Proposed federated learning to detect DDoS in the IIoT	Low mitigation response time with high mitigation accuracy	High accuracy and low response time	Needs more tests to implement in real-world IIoT settings	2020
[64]	Network security in IoT systems	NSL-KDD	Proposed a two-stage hybrid using ML algorithms and a genetic algorithm	Ensemble classifier performed better with 99.8% accuracy	Can reduce cyberattacks and improve security	Needs more testing on actual IoT networks	2021
[65]	Malicious bot-IoT traffic in IoT networks	Bot-IoT	Proposed a novel metric called CorrAUC based on the AUC metric	The model was effective and achieved 96% accuracy	High accuracy in detecting malicious traffic	Not scalable and needs more training data	2021
[66]	Identifying intrusions in network activity	CSE-CIC-IDS2018	Used DT, EF, KNN, and GNB ML models	RF scored the highest F1 score of 89.5% with a precision of 99.3%	Early detection of malicious attacks; efficient and accurate	Needs better parameters and a wider dataset range	2022
[67]	IoT malware identification	IoT-23, LITNET-2020, and NetML-2020	Used both ML and DL algorithms on datasets to detect malware on datasets	RF achieved the highest accuracy score of 96%	Exhibits high accuracy in classifying malware	Management of large datasets	2022
[68]	Mitigating IoT-Botnet attacks using NIDS for the IoT	CICIoT2023	Proposed solution with ML models to detect Botnet attacks	DT was most accurate with a 99.17% score, followed by RF and KNN	A wide-ranging dataset was used	Computationally complex	2023
[42]	Detecting DDoS attacks in SDN IoT	Private data	NB, DT, SVM model classifiers used to test attacks in the IoT	DT achieved 98.1% accuracy, outperforming other models	Reduces the impact of DDoS attacks	Enhanced the system	2023
[69]	Detecting cyberattacks in Industrial IoT (IIoT) scenarios with hybrid ML models	DS2OS	Proposed framework combines different ML models to form an HML	The models scored an accuracy of 99.8% in detecting abnormal traffic	Use of a comprehensive dataset and high accuracy of detection	The framework is computationally heavy	2023
[70]	Using ML models to prevent security threats in the IoT	-	Proposed the DT model to classify abnormal data	Successful in detecting and classifying abnormal data	Works in maintaining security	No specific accuracy or performance rates are mentioned in the paper	2023
[71]	Cyberattacks and IDSs in IoT networks	IoT-23 and IoT Network Intrusion	Compared different ML models—RF, DT, NB, MLP, and KNN	RF and DT performed best with an accuracy of 99.9% each	Highly accurate in classifying malicious activity	Needs larger datasets and improved accuracy of the models	2023
[72]	Detecting DoS attacks with datasets	IoTID20	Features selected via a GA and CFS trained with KNN, DT, RF, SVM classifiers	DT and RF achieved 100% accuracy with the GA	Used recent and real-time data	Lack of scalability	2024
[73]	Detecting attacks using ML	Smart Home Testbed, UNSW-NB15, CIFAR-10, Kitsune, Bot-IoT, NSL-KDD	Used NV, RF, DT, and SVM models to detect adversary attacks in the IoT	RF showed the most resilience- accuracy dropped only 21%	Showed innovative detection methods	Lack of a balanced dataset	2024
[74]	Detection of malware in the IoT	UNSW-NB15	Used ML models—LR, KNN, DT, ET, RF, and MLP	ET achieved 99.98% accuracy	Used large and diverse dataset	Difficult to detect a zero-day attack	2024

## 4. Deep Learning—IoT Network Anomaly Detection

**Figure 3:**
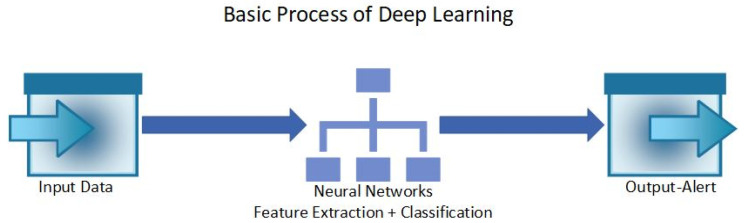
The basic process of a deep learning algorithm that is used to detect anomalies in the IoT.

Deep learning (DL) involves the use of Neural Network architecture to detect anomalies in a system. Neural Networks are designed to mimic the human brain. where each layer processes the input data [75]. Due to the use of Neural Networks, DL can process large and complex data to extract relevant information [76]. DL can be used as supervised, unsupervised, or semi-supervised learning [76]. Different types of DL models include Convolutional Neural Networks (CNNs), Recurrent Neural Networks (RNNs), Temporal Convolutional Networks (TCNs), Autoencoders, Generative Adversarial Networks (GANs), Deep Belief Networks (DBNs), Long Short-Term Memory (LSTM) Neural Networks, and more [77,78]. Compared to ML models, it is mentioned that DL models are better at detecting anomalies that were previously not detected [76] and are more capable of analyzing large and complex datasets, which makes them suitable for the IoT [75]. Hence, DL models are said to be better suited for security and privacy maintenance in IoT systems [79]. In addition to anomaly detection, other benefits of DL models include predictive analysis, automation, improved accuracy, scalability, efficiency, and more [77,79,80]. Figure 3 shows the basic process of a DL-based algorithm’s function, where the input is the data from IoT environments and the output is a binary alert system that informs the user whether the data are anomalous or normal.

Anomaly detection with DL is categorized into the detection of anomalies in the IoT which is presented in a summarized format in Table 4, and the detection of attacks which is presented in Table 5. Attacks in this study are considered anomalies; however, the two are divided systematically in order to categorize them.

### 4.1. Anomaly Detection

To enhance security in the IoT of smart cities, the study in [81] suggests an IoT detection system for intrusions that makes use of deep migration learning. Utilizing a large number of network connection records from the KDD CUP 99 dataset, the system detects anomalies and implements the appropriate corrective actions. At 99.78% accuracy, 0.22% false alarms, and 98.99% precision, the proposed method performs better than established techniques like Extreme Learning Machine (ELM) and Backpropagation (BP). It demonstrates increased efficiency, a decreased false positive rate, and a greater detection rate. It may, however, exhibit reduced classification accuracy during compression, necessitating more research on classifier adjustment in real time. This study offers a viable strategy for enhancing security in intelligent urban areas. 

To solve scalability concerns, the study in [82] proposes a framework for anomaly identification in IoT communications. For this, traffic logs are distributed across fog nodes for parallel learning in the framework using vector convolutional DL (VCDL). An evaluation was conducted using the Bot-IoT dataset, which consists of attack and regular traffic logs from IoT smart home devices. The framework was put into practice using Keras on a Theano package, Apache Spark, and an Intel Core i7-6700 processor. The results indicated that, in comparison to other systems like SVMs, LSTM Networks, and RNNs, the framework facilitated distributed anomaly detection with a lower detection time and showed notable performance benefits. With an accuracy score of 99.7%, the model outperformed all other models. Nevertheless, there are certain drawbacks to this study, including class imbalance issues that may result in lower detection accuracy in multi-class classification.

Additionally, another study [36] conducted a survey on IDSs in IoT networks and the limitations of conventional systems. It explored various DL methods including Convolutional Neural Networks (CNNs), Long Short-Term Memory Networks (LSTM Networks), and Recurrent Neural Networks (RNNs). It also explored various datasets that are used with DL models, such as UNSW-NB15, NSL-KDD, UNB ISCX 2012, and KDD CUP 99, which are used often in experiments as they contain raw data. It also surveyed various studies of IDSs using DL models with various datasets. From this survey, Deep Neural Networks (DNNs), Forward Neural Networks (FNNs), and Recurrent Neural Networks (RNNs) were shown to have the best detection, with an average accuracy of 99.7%.

A new system was proposed by the study in [83] on deep transfer learning-based intrusion detection system architecture to address the shortcomings of traditional network intrusion detection techniques in managing modern network infrastructure, including real-time processing and increasing network traffic complexity. The proposed system trains an algorithm on a source domain using more data and processing power, then moves to an intended domain using less data and processing power. The system classifies network traffic as malicious or legitimate. The proposed system was tested using the UNSW-NB15 dataset and used various DL methods, including a hybrid CNN-LSTM model, Long Short-Term Memory Networks, and Convolutional Neural Networks. The results showed that the proposed deep transfer learning-based design outperformed a state-of-the-art IDS, achieving high accuracy scores of 98.43% in both the source and target domains. The architecture’s combination of CNNs and LSTM Networks proved effective, allowing it to process network traffic in real time. The research also aims to further validate the proposed framework with more datasets.

Regarding Industry 4.0 applications based on the IoT, one study [84] addressed the problem of real-time anomaly detection in time-series data. It offered a prediction-based approach for anomaly identification in time-series data by utilizing a multi-source prediction module and a cutting-edge detection technique. Two real-world datasets, Numenta Anomaly Benchmark (NAB) and Yahoo Webscope, were used to evaluate this approach. The proposed system works by using time-series data as the input, using a multi-source prediction module—Prediction-Driven Anomaly Detection (PDAD-SID)—to improve model reliability by considering the ensemble of successive predictions and giving each prediction source a probabilistic score. The output is a probability for an incoming anomaly record using the SID metric and using predicted sequences to assess variations of a real sequence from anticipated sequences in real time. The results showed that the proposed PDAD-SID method outperformed the current anomaly detection techniques in the AUC metric (overall performance) of 92.6%, outperforming cutting-edge techniques like LSTM Networks in terms of TPR, FPR, and AUC-ROC.

Examining information security in the context of the IoT, the research in [85] emphasized the significance of an effective IDS. The research suggested a DL model for identifying irregularities in IoT networks, evaluated its performance on many datasets, and contrasted it with rule-based systems and traditional ML techniques. The suggested model classifies normal and abnormal traffic using recursive feature removal and transfer learning applied to network traffic data as the input. The model was trained and tested on four datasets, comparing its performance to rule-based systems and conventional ML methods. The CNN1D, CNN2D, and CNN3D models achieved minimum detection rates of 99.74%, 99.42%, and 99.03% for the BoT-IoT, MQTT-IoT-IDS2020, IoT-23, and IoT-DS-2 datasets, respectively. The results showed that the DL-based model performed better than conventional ML methods and rule-based systems in terms of precision, recall, accuracy, and F1 score. However, more research is recommended to test the models with real data.

With regards to IDSs, the study in [86] proposed an IDS for detecting anomalies in IoT networks using DL models. The proposed system uses pre-processed network traffic data to train a deep Convolutional Neural Network (CNN) to distinguish between regular and abnormal traffic. The model outputs a binary-based classification outcome. The experimental setup used two datasets: NSL-KDD and UNSW-NB15. The model’s performance was assessed using precision, recall, accuracy, and F1 score measures. The results showed that the suggested method performed better than conventional ML models in terms of F1 score, accuracy, precision, and recall. On the NSL-KDD dataset, the system achieved 99.8% accuracy and a 0.998 F1 score, while on the UNSW-NB15 dataset, it achieved 99.9% accuracy and a 0.999 F1 score.

Similarly, another study [87] explored the limitations of current IDSs in IoT networks, focusing on the heterogeneity of traffic streams and disregarding spatial and temporal correlations. The paper proposed a hierarchical-based semi-supervised training method, called SS-Deep-ID, that considers the sequential characteristics of IoT traffic data, incorporating a multiscale residual temporal convolutional module and optimized traffic attention mechanism. The system’s output predicts whether an incursion is detected in the traffic data. The method was tested on two datasets, CIC-IDS2017, and CIC-IDS2018, and the results showed improved precision, accuracy, recall, and F1 score compared to existing approaches, with an accuracy above 99% and F1 measure between 98% and 99%. Hence, the study concluded that this hierarchical semi-supervised method enhances network performance by considering the sequential properties of IoT traffic data. Future work is recommended to make the model detect anomalies in real time and make it general and scalable so that it works in various scenarios.

Regarding IDSs, another study [88] worked on detecting intrusions in IoT devices using an intrusion detection system built on the SDN architecture. The proposed system uses network traffic data from IoT devices to distinguish between malicious and legitimate traffic using a DL LSTM algorithm. The system compares data flows and actions within the network to address security issues. The experimental setup used the CSE-CIC-IDS2018 dataset to train the LSTM model. The results showed that the proposed system of IDSIoT-SDL performs better in accuracy, sensitivity, and false positive rate than current methods. The four security parameters considered were false positive rate, detection rate, specificity, and sensitivity. The IDSIoT-SDL simulation results showed 1775 true positives, 212 true negatives, 12 false positives, and 7 false negatives, and an accuracy of 99.05%. This paper provides a novel method of combining SDN and DL methods for IDSs in IoT traffic. Moreover, further research can be conducted with other DL models and in real environments to enhance the proposed model.

As stated previously, IoT networks and devices are susceptible to intrusions that try to undermine data integrity and service availability. One study [76] examined these security flaws. This study suggested a DL-enabled solution that leverages the network traffic of IoT devices as input data (DS2OS) to identify anomalies in IoT security. The system simulates the probability distribution of normal behavior and recognizes normal network behavior using a Deep Neural Network (DNN). The input is labeled as anomalous if the error surpasses a set threshold. A binary classification of regular or anomalous traffic is the system’s output. The system was evaluated against other alternatives, such as rule-based and signature-based IDSs, utilizing actual traffic data from an IoT network-connected smart house. The accuracy of the method was 99.8%, demonstrating its potential for dependable security in decision-support systems. This study, while highlighting an accurate and efficient technique of anomaly detection, also suggests further research with more datasets and real environments.

Another paper [89] suggested a DL-based intrusion detection system to enhance IoT device security in smart cities. This system uses network traffic data from IoT devices, which are pre-processed and loaded into a DL and ML model to detect intrusions. The proposed model used the Minority Oversampling Technique (SMOTE) with a voting classifier and the dataset ToN-IoT Telemetry. The system outputs a binary classification of regular or anomalous traffic. The experimental setup used an IoT device network traffic dataset for training and testing. The system was compared to existing options like rule-based and conventional ML-based IDSs. The results showed that the proposed system performed better than current solutions in precision, recall, accuracy, and F1 score, with the voting classifier with SMOTE achieving 99.7% accuracy. In addition, the system demonstrated a lower rate of false positives and false negatives than the existing systems. Further studies are recommended with more DL models and varied datasets to achieve a comprehensive analysis.

The problem of false alarms in IDSs, which can lead to alert fatigue and make it challenging to identify actual security incidents, is the subject of the research in [90]. The authors provide a technique for increasing the accuracy of false alert detection by combining DL and ML. This is carried out by inputting the result of hidden layers of DNNs into ML models such as the DT, GNB, KNN, RF, and AB models. Using a traffic log dataset, the proposed model was used to detect false alert rates, and its detection was compared to that of the conventional ML models alone. Combining the DNN with the RF gave the best accuracy, at an average of 96.7%. The study also notes that the use of DL and ML models improved the detection of false alerts and recommends this approach for further research. However, the study is limited, as it uses one dataset and does not compare its findings to those of other approaches.

Another study’s [91] goal was to create an industrial IoT intrusion detection system. The WUSTL-IIOT-2021 dataset was used by this system. In this study, class imbalances and irrelevant characteristics were addressed in deep IIoT scenarios through the employment of a deep-learning classifier to analyze features. The solution solved the dataset imbalance problem and performed better than existing IDSs. However, its applicability to different IIoT scenarios could be limited by its dependence on a particular dataset.

Based on a CNN-based IDS, the study in [92] examined the difficulties associated with CNN-based detection of intrusions in diverse network environments, as well as how CNNs may be used for the extraction and classification of features and how well they perform when measured with the right metrics. The study divided CNN-based intrusion detection methods into several classes based on the WUSTL-IIOT-2021 dataset it used. The method achieved an accuracy of more than 99%, with a 0.069% false positive rate. This study states that the model outperformed other models in the same domain and that further research is required to make its classification of anomalies more sophisticated than a binary result.

The difficulty of deciphering Deep Neural Network judgments in detection systems for intrusion is discussed in [93]. This paper suggests a CNN model and hybrid CNN models with LSTM or Autoencoder methodology for understanding cyberattacks in the NSL-KDD and CICIDS2017 datasets. The hybrid LSTM model with 1D-CNN showed the best accuracy, at 98.02% with the CICIDS2017 dataset and 89.93% with NSL-KDD. This framework improves human comprehension, produces very precise results, and gives clear descriptions of the CNN detection technique. Nevertheless, it has drawbacks in real-world deployment, explainability strategies, model optimization, and dataset selection.

Using DL algorithms, the research in [94] aimed to detect anomalies in IoT data. The proposed system receives time-series data from IoT devices, pre-processes it to eliminate outliers and noise, and uses a DL model to identify anomalies. The model, which is either a Temporal Convolutional Network (TCN), Long Short-Term Memory (LSTM) Network, BI-LSTM, or CuDNN-LSTM, was trained on pre-processed data from Secure Water Treatment (SWaT) to identify patterns and abnormalities. The experimental setup involved pre-processing data from publicly accessible datasets and training and evaluating DL models. The results showed that the Root Mean Square Error (RMSE) for the prediction accuracy of CuDNN-LSTM was an average of 0.042, which is more accurate, but it required more training time. While TCN had an average RMSE of 0.161, it required less training time. The authors also provided evidence of their method’s effectiveness in identifying irregularities in actual IoT data, with larger timestamped values resulting in longer training times but better model accuracy performance. For better analysis, the study recommends further research with different datasets to compare between the two methods.

Furthermore, another research’s [95] objective included improving IDSs in IoT networks using DL. This project addressed the difficulty of interpreting the judgments made by AI algorithms used in detecting intrusions in IoT networks. The research presented an implementation of a deep SHAP with a CNN for explaining IDS output in IoT networks and suggested a structure for the global and local justification of IDSs based on artificial intelligence in IoT and IoV communication networks. The proposed system processes network traffic data from IoT-enabled transportation networks with the ToN_IoT dataset, and uses the CNN deep SHAP approach to understand its output and offer regional and global justifications for its choices. The proposed system achieved a higher accuracy of 99.15% and an F1 score of 98.83% compared to previous SHAP approaches and conventional ML-based and DL-based IDSs. However a drawback of this method is that SHAP is computationally heavy, is vulnerable to attacks, and is costly, which might hinder its implementation in the IoT environment.

For IoT-enabled smart cities, the paper in [96] suggests two-tiered detection of intrusions based on the anomaly method. This methodology enhances system efficiency and chooses the best features for IoT IDSs by utilizing DL techniques and lightweight ML algorithms, namely the combination of the lightweight GBC with a CNN model. Employing the UNSW-NB15 dataset, the study verified the proposed approach and produced competitive results when compared to other methods (such as CNN-BiLSTM), with an accuracy of 99.85%. The benefits of this approach include improved feature selection and a collaborative IDS architecture. However, limitations in resources and validation in real-world settings could be drawbacks.

Providing a targeted approach for identifying anomalies in network activity, another study [97] researched detecting cyberattacks in distributed and heterogeneous fog computing environments. The proposed system operates by utilizing data on network traffic from several fog ecosystem edge units. It employs a federated deep Q-learning network (FDQN) method, with local learning and global learning phases. Edge units learn a unique deep reinforcement learning framework using local data and share their models through aggregation points. The system outputs a list of abnormalities in network flow. The experimental setup involved a set of network traffic data from the NS-3 network simulator, which was dispersed among edge units to employ the suggested service-based FDQN technique to identify network traffic irregularities, and two services were focused on for the data—DNS and HTTP. The results showed that the suggested solution performed better than current options in terms of resource usage and detection accuracy. More research is recommended on sophisticated DL methods to explore better detection techniques.

Using the KDD99 dataset, a novel DL technique for identifying anomalies in IoT devices is presented in [98]. A CNN and LSTM Network were combined to form a C^2^-LSTM model in the suggested architecture to handle large amounts of data with a high degree of sensitivity. The KDD99 dataset was used in this study for measuring performance. When measured against other DL implementations currently in use, namely the CNN, LSTM, and C-LSTM models, the proposed model yielded improved accuracy, precision, and recall, with an accuracy of about 99%. Nevertheless, it presents problems related to obsolescence, as pre-processing data can lead to latency and needs more investigation.

In [99], DIS-IoT—a method for intrusion detection in IoT environments—is presented. It integrates four DL models: an LSTM-based model, a CNN-based model, a Deep Neural Network (DNN), and a shallow Multilayer Perceptron (MLP). The models were evaluated with CICIDS2017, ToN_IoT, and SWaT datasets. This study indicated that DIS-IoT achieved good scores in both multi-class and binary classification for precision, recall, accuracy, and F1 score using three open-source datasets. With the ToN_IoT dataset, the proposed DIS-IoT achieved 99.6% accuracy; with the CICIDS2017 dataset, the model achieved 98.7% accuracy; and with SwaT, it achieved 99.7% accuracy. For future research, it is recommended that this proposed model be tested with actual IoT devices.

### 4.2. Attack-Based Anomaly Detection

The application of DL for distributed fog-level cyberattack detection in IoT networks is covered in [100]. A Deep Neural Network was evaluated and validated in this study with the NSL-KDD intrusion dataset. In the study, the efficacy of the Deep Neural Network model achieved a high accuracy of 99.2% with a two-class model and 98.2% accuracy with a four-class model. Improved scalability, parameter sharing, and precision are among the benefits of this approach. Longer training times, complicated implementation, and a need for huge training datasets are its drawbacks. This study emphasizes that DL might improve the detection of cyberattacks in IoT networks, while it might require longer training times and a larger dataset than ML models. 

The application of DL approaches to improve security in SDN-based IoT architecture is explored in [101]. This study develops an IDS employing Restricted Boltzmann Machines (RBMs) for the detection of anomalies and attacks in the network using the widely used KDD99 dataset. The system’s precision rate, above 94%, shows the promise of DL for IoT networks. The study sheds light on how DL models may be used to detect network anomalies, which could improve cybersecurity in IoT settings. It does not, however, provide comprehensive information on performance and scalability in large-scale IoT systems.

The study in [102] addresses the problem of identifying malicious activity in IoT backbone networks, emphasizing the requirement for effective large data-processing tools and detection algorithms. A suggested framework that makes use of the Keras DL Library is tested in this study using the UNSW-NB15 and NSL-KDD99 datasets. Convolutional Neural Networks (CNNs), Autoencoders, Deep Neural Networks (DNNs), and Multilayer Perceptrons (MLPs) are the four DL models used by the framework. The results show that the DNN model outperformed the MLP model, which had an accuracy rate of 98.96%, with a rate of 99.24%. The study offers high F1 values and accuracy in identifying anomalies in IoT environments, but it also points out that one potential limitation of DL models may be their complexity, which makes them computationally demanding for use in real-world IoT systems.

The research in [103] offers a distributed DL framework that may be used to detect and mitigate Botnet and phishing attempts, thus improving the security of IoT devices. The PhishTank, OpenPhish, Curlie, and “Detection of IoT botnet attacks N_BaIoT” databases are among the datasets used in this study. To identify and prevent attacks at their point of origin, the proposed framework makes use of a Long Short-Term Memory (LSTM) Neural Network. With 94.3% accuracy and a 93.58% F1 score, the IoT microsecurity addition effectively identified phishing assaults. An accuracy of 94.80% was attained by the LSTM algorithm used for Botnet attack detection. The study provides benefits like distributed attack detection by integrating CNN and LSTM models, which achieved high accuracy but could be complex when implemented in a real-world IoT environment.

The study in [104] offers a method for identifying and stopping intrusions in IoT networks. The authors developed a hybrid DL architecture for attack detection that incorporated Long Short-Term Memory (LSTM) and Convolutional Neural Network (CNN) models that were tested on the IoT-23 dataset. In this experimental scenario, the model was trained using the dataset, yielding a 96% detection accuracy and a 97% recall value for IoT threats. The study showcased the use of the CNN and LSTM models together and achieved improved accuracy and efficiency through such, but it also notes certain drawbacks, namely its reliance on a single dataset and the requirement of additional validation across a variety of datasets.

Considering DDoS attacks, the research in [105] aimed to protect IoT devices from attacks like distributed denial-of-service (DDoS) and denial-of-service (DoS) attacks using the DeL-IoT deep ensemble learning method using SDN. The proposed system uses data from IoT devices and apps, extracts important aspects, and uses behavioral analysis to determine labeling. The detection module tracks traffic and system parameters, and the learning module uses a deep ensemble learning technique to find anomalies. The experimental setup involved creating various attacks and gathering metrics. The results showed that the DeL-IoT technique outperformed state-of-the-art ML-based methods in detecting anomalies, with a 99.8% detection rate on testbeds and a 99.9% rate on benchmark datasets.

In [106], DL methods are used to detect brute-force assaults on IoT networks. The study proposed using a supervised DL model to detect attacks in the MQTT-IoT-IDS2020 dataset. Researchers used the Deeplearning4j library in Java to integrate the DL model on which the dataset would be used. The study stated that the DL classifier showed high accuracy, with an accuracy rate of 99.6% on bi-flow and 99.7% accuracy on uni-flow features using the MQTT-IoT-IDS2020 dataset. The study showed robust techniques for detecting attacks in the IoT using DL models; however, the use of one dataset might restrict the generalizability and ignore other kinds of attacks.

The paper in [107] uses the Edge-IIoTset dataset, containing a variety of cyberattacks, to propose an effective methodology for IIoT intrusion detection. The deep transfer learning (DTL) framework makes use of bootstrap aggregation ensemble techniques, Convolutional Neural Networks (CNNs), and genetic algorithms (GAs). With fourteen types of cyberattacks predicted, it exceeds modern systems for intrusion detection in accuracy with a score of 100%. While to increase its real-time detection, scalability, and resilience, more research is needed, the proposed framework outperformed other DTL models.

In [108], cyberattacks on IoT networks were examined, along with the necessity of effective security protocols. This study proposed the use of several DL models, namely Feed Forward Neural Network (FFNN), Long Short Term Memory (LSTM), and Random Neural Network (RandNN) models, that were each trained on the CIC IoT 2022 dataset. Using data from IoT sensors and devices, the proposed system inputs and outputs anomalies and cyber threats within the IoT through DL models for binary and multi-class classification. An IoT device and a dataset of network traffic generated by sensors were employed in the experimental setup. The FFNN scored an accuracy of 99.93%, the LSTM model scored 99.7%, and the RandNN achieved 96.42% accuracy, making the FFNN the most accurate among them. The proposed IDS can extract and classify features in a versatile way, making it efficient in detecting cyberattacks in the IoT. For future work, exploration of more DL models is recommended to build a sophisticated system for implementation in IoT environments.

**Table 4:** Summary of the DL-based literature for anomaly detection in the IoT environment.

Ref.	Problem Addressed	Dataset	Proposed Solution	Results Obtained	Advantages	Disadvantages	Year
[81]	DL-based anomaly detection in smart cities	KDD CUP 99	Deep mitigation learning proposed	The model achieved 99.78% accuracy and outperformed BP and ELM	Can enhance security in urban areas	Classification accuracy reduced during compression	2019
[82]	DL-based anomaly detection	BoT-IoT	VCDL model proposed	Achieved 99.7% accuracy	Outperformed other models	Class imbalance issues	2020
[36]	DL-based IDSs in IoT networks	UNSW—NB15, NSL-KDD, UNB ISCX 2012, and KDD CUP 99	Surveys various DL models in studies—DNN, CNN, RNN, FNN, and more	DNN, FNN, and RNN performed best with 99.7% accuracy	Showcased several DL model results on various datasets	Further study is needed to explore more DL models with other datasets	2021
[83]	DL-based IDSs	UNSW-15	DL-based CNN-LSTM model proposed	Achieved an accuracy of 98.43% across all domains	Detected anomalies in resource-constrained domains	Needs more research with more varied datasets	2021
[84]	Real-time anomaly detection in time-series data	NAB and the Yahoo Webscope	PDAD-SID model proposed to detect anomalies	Outperformed other models like LSTM with an AUC score of 92.6%	Can be applied to various Industry 4.0 applications	Needs testing with more complex time-series data types	2021
[85]	DL-based anomaly detection in the IoT	BoT-IoT, MQTT-IoT-IDS2020, IoT-23, IoT-DS-1, and IoT-DS-2	CNN1D, CNN2D, and CNN3D models proposed	All models achieved an accuracy >99% for all datasets	Proposed DL models outperformed other models	Limited datasets and lack of actual testing	2022
[86]	DL-based IDSs	NSL-KDD and UNSW-NB15	CNN models proposed to detect anomalies in datasets	The model achieved an average of 99% accuracy on both datasets	Efficient in finding anomalies in IoT networks	Needs more testing with larger and real datasets	2022
[87]	Heterogeneity of traffic in IoT devices	CIC-IDS2017 and CIC-IDS2018	Semi-supervised method proposed called SS-Deep-ID	Achieved an accuracy >99% with the datasets	Integrated into fog-enabled IoT networks	Computational overhead is significant	2022
[88]	SDN and DL for IDSs in IoT	CSE-CIC-IDS2018	SDN architecture IDSIoT-SDL used with the LSTM DL model	The model had an accuracy of 99.05% and 212 true negatives	High accuracy and low false positive rates	Needs testing with DL models and in real environments	2022
[76]	DL-enabled anomaly identification	DS2OS	DNN DL-based model proposed	Achieved an accuracy of 99.8%	Accurate and efficient anomaly detection	Needs testing with more datasets and real-world situations	2022
[89]	Anomaly detection in smart cities	ToN-IoT Telemetry	Compared DL and ML-based models with the dataset	The voting classifier with SMOTE achieved 99.7% accuracy	Compared to many learning models	Needs more testing with more varied datasets	2022
[90]	False alert detection in IDSs in the IoT	Traffic log	Combined ML and DNN to detect false alerts	DNN with RF had an accuracy of 96.7%, which was higher than other ML models	Used real alert records from traffic log data	Needs testing with more datasets and comparison to other models	2022
[91]	IDSs in the IIoT	WUSTL-IIOT-2021	Used DL models with network flow data for an IDS	Achieved a 99% accuracy rating	Successful in handling class imbalance in the dataset	Needs more testing with more varied datasets	2023
[92]	DL-based IDSs in the IIoT	WUSTL-IIOT-2021	DL models applied to the dataset to detect anomalies	DeepIIoT achieved >99% accuracy	Higher accuracy than others in the IIoT	Better classification of anomalies could be achieved	2023
[93]	Interpreting DL decisions with IDSs in the IoT	CICIDS2017 and NSL-KDD	CNN models and a hybrid CNN model with LSTM and Autoencoder	LSTM with 1D-CNN showed 98.02% accuracy with CICIDS2017	Thorough study of CNNs and other DL-models	Needs more varied datasets, model optimization	2023
[94]	Detect anomalies in IoT data using DL techniques	SWaT (Secure Water Treatment)	Compared TCN, LSTM, BI-LSTM, and CuDNN-LSTM on SWaT	The average RMSE of CuDNN-LSTM was 0.042, with more time, and TCN was 0.161, with less time	Effective in detecting anomalies	Needs testing with different datasets	2023
[95]	Anomaly detection in IDSs with DL	ToN_IoT	Implemented deep SHAP with the CNN model	Achieved accuracy of 99.15% and F1 score of 98.83%	Increased accuracy and F1 score than previous SHAP	SHAP is computationally heavy and costly	2023
[96]	Anomaly detection with DL and ML	UNSW-NB15	Proposed two-tier classification with GBC and CNNs	Achieved an accuracy of 99.85%	Employs ML and DL collaboration	Needs further validation in a real-world setting	2023
[97]	Anomaly detection by federated DL	UNSW-NB15	FDQN used on the dataset to detect anomalies	Performed better in resource usage and detection accuracy	Scalable, versatile, and outperformed other models	The exact values of metrics are not mentioned	2023
[98]	IDSs in the IoT in Industry 4.0 applications	KDD99	Combined a CNN with LSTM to form C^2^-LSTM	Achieved high accuracy, precision, recall, and AUC score	Extracted temporal and spatial features separately	An old dataset was used. Testing is needed with a newer dataset	2023
[99]	IDSs in the IoT with DL-based models	ToN_IoT, CICIDS2017, and SWaT	Proposed a stacking ensemble of DL models named DIS-IoT	Accuracy score with ToN_IoT was 99.6%, with CICIDS2017 was 98.7%, and with SWaT was 99.7%	Outperformed other models in all metrics	Needs testing with real IoT devices	2024

**Table 5:** Summary of the DL-based literature for detecting attacks and anomalies in the IoT environment.

Ref.	Problem Addressed	Dataset	Proposed Solution	Results Obtained	Advantages	Disadvantages	Year
[100]	DL-based cyberattack detection	NSL-KDD	DNN proposed	Accuracy score of 99.2% with a two-class model	Improved detection of cyberattacks	Longer training time and needs a large dataset	2018
[101]	IDSs for attack detection	KDD99	RBM employed for detection	A precision rate of 94% was achieved	The ability of DL models to detect an attack	Comprehensive results not mentioned	2018
[102]	Detecting malicious activity in the IoT with DL	UNSW-NB15 and NSL-KDD99	Four DL models were used—CNN, DNN, MLP, and Autoencoder	DNN outperformed others with an accuracy of 99.24%	High accuracy and F1 results achieved	Complex model and computationally heavy	2019
[103]	Botnet and phishing attacks in the IoT	PhishTan, OpenPhish, Curlie	LSTM neural network proposed	Accuracy with botnet attack was 94.8%; accuracy with phishing was 94.3%	Integrated CNN and LSTM models	Complex to implement in a real environment	2020
[104]	Identifying attacks in the IoT	IoT-23	Hybrid DL model of CNN and LSTM	Achieved a detection accuracy of 96%	Improved accuracy and efficiency	Needs testing with more datasets	2021
[105]	Detecting DDoS and DoS attacks in the IoT	Collected data and N-BaIoT	DeL-IoT deep ensemble learning model	Outperformed ML methods with a 99.8% detection rate	Provides accuracy and scalability	More tests are needed with varied datasets	2021
[106]	Brute-force attacks in the IoT	MQTT-IoT-IDS2020	Featured bi-flow and uni-flow DL-based models	The bi-flow feature had 99.6% accuracy and the uni-flow feature had 99.7% accuracy	High accuracy in detection	Needs more datasets for testing	2023
[107]	Cyberattacks and device profiling in the IoT	Edge-IIoTset	DTL model with a CNN, GA, and aggregation ensemble	Achieved 100% accuracy and detected various cyberattacks	Incorporated a realistic dataset	Needs more research for scalability and real-time detection	2023
[108]	Detecting cyberattacks with DL	CIC IoT 2022	FFNN, LSTM, and RandNN were used to test the dataset	Accuracy score of FFNN was 99.93%, of LSTM was 99.7%, of was RandNN 96.42%	Versatile extraction and classification features	Optimization needed with more diverse datasets	2023

Conclusively, using a DL-based algorithm shows promise for the accurate detection of anomalies. However, the challenge with DL-based algorithms is that they need large and high-quality datasets, are computationally heavy, and require time to train. Moreover, the complexity of DL algorithms makes it difficult to source the reason or pathway of the decision-making process.

## 5. Research Summary

**Figure 4:**
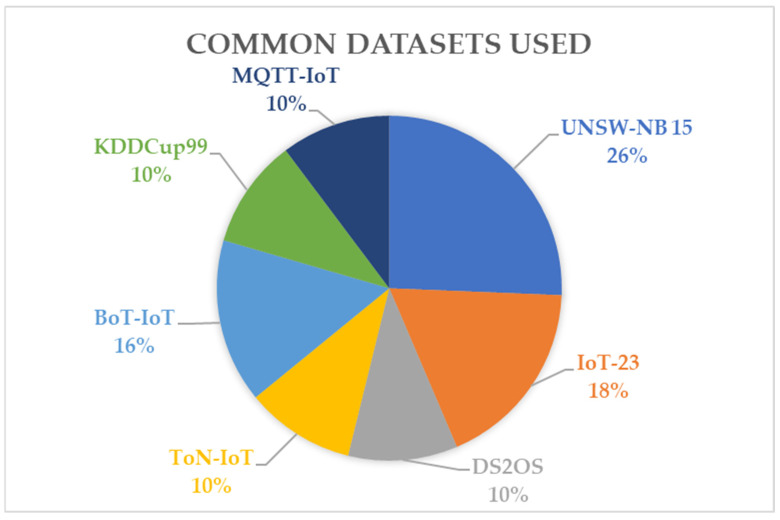
Most common datasets used in the research papers analyzed in this study.

For the ML-based studies, the model most cited to have the highest accuracy is the Random Forest (RF) model, which was cited about twelve times to have the highest accuracy [49,50]. The models least cited to have a high accuracy are the ANN, GBM, and RT models. The ranged uses of RFs over the years makes it an accurate ML model for detecting anomalies and attacks. Most of the studies share a common drawback, which is the need for more varied datasets to validate the proposed models [59,64,71]. This is followed by the drawback that the models can be computationally heavy for IoT systems [69]. Figure 4 includes a summary of the datasets most used in the studies considered for this study. According to Figure 4 among all of the datasets, UNSW-NB 15 and IoT-23 appear to be the most used datasets for both ML and DL model testing [56,63].

For the DL-based studies, the model most cited with the highest accuracy is the Long Short-Term Memory (LSTM) Neural Network and its hybrid [85,89], which appeared about seven times, followed by the Conventional Neural Network (CNN) [87] and its hybrid [84], which appeared about six times, followed by the Deep Neural Network (DNN) [36]. Hence, from the literature, LSTM, CNN, and DNN models appear to be the most accurate DL models in the detection of anomalies and attacks. The most common drawbacks for DL-based models appears to be the need for more varied datasets [36] and larger datasets [86], and the computational complexity of the models [88,104].

## 6. Research Gaps

The current work on detecting anomalies in IoT networks using ML techniques has several limitations and areas for improvement. These include the inaccuracy of current algorithms, the lack of consideration of adversarial attacks, the complexity and variability of IoT data, the lack of privacy and security issues, and the insufficient comprehensiveness of frameworks for evaluating the performance of ML and DL algorithms. The literature also fails to consider the trade-offs between accuracy, efficiency, and scalability in ML-based anomaly detection systems. The methods for visualizing and interpreting anomaly detection results are not user-friendly for non-experts. Novel approaches to anomaly detection, such as DL and reinforcement learning, are not explored thoroughly. Moreover, the current evaluation methods do not consider the heterogeneity and variability of IoT devices and networks, and the impact of network topology and architecture on the performance of ML-based anomaly detection systems.

## 7. Areas for Improvement

The existing work on detecting anomalies in IoT networks using ML and DL techniques needs improvement. The possible improvements include developing more accurate and efficient algorithms, exploring new approaches such as reinforcement learning, the use of blockchain technology to enhance security and privacy, improving data collection and preprocessing methods, conducting extensive experiments to evaluate algorithm performance, developing user-friendly interfaces and visualization tools, and exploring real-time anomaly detection. These improvements aim to enhance the security and privacy of IoT networks while ensuring the safety of the devices.

This is significant as the IoT is increasingly being integrated into different environments, such as smart homes, smart governments, smart cities, agriculture, industries, and more. With this integration comes the risk of attacks and adversarial traffic, which may cause harm to the device or the entire system. These attacks can be used for ransomware and data breaches. Hence, developing intrusion detection methods that can detect new attacks, take less time to detect anomalies, and are dynamic is essential in keeping up with the growing use of IoT systems. This can be achieved through AI with ML and DL models. Moreover, the use of AI to detect anomalies remains relatively recent and needs more attention to develop thorough policies and standards to enforce guidelines on the use of AI models for IDSs. Therefore, further research in this field is crucial to develop robust and scalable security and privacy measures in IoT systems.

## 8. Conclusions

This paper addresses the difficulties in locating intrusions and anomalies in IoT systems, which could lead to a breakdown of the system. The justification for this research is due to increasing attacks in IoT systems. The paper then presents a comprehensive review of the most recent work on machine learning-based and deep learning-based anomaly detection schemes for IoT networks. All of studies from the literature in this review are summarized in tabular format. Overall, most papers suggested novel systems for detecting intrusions in IoT systems, which were then compared with existing models using various performance and security metrics to determine the suggested models’ efficiency and accuracy.

Most of the research provides an introductory framework for anomaly detection, which is suggested by the researchers to further develop. The way to develop these existing systems is to firstly use more varied datasets to train the AI systems. Other ways to improve the systems are through testing them in real-time and in different environments. The systems also need to be made scalable and sophisticated to successfully detect anomalies in IoT systems in real-world settings. Regarding DL-based algorithms, more research is needed to integrate them in IoT environments, as DL algorithms are computationally heavy.

## Figures and Tables

**Table 1:** Summary of attacks in the IoT mentioned in the literature.

Reference	Attacks
[12]	Spoofing, Sleep deprivation, Replay, Session hijacking
[13]	Spyware, Trojans, Sinkhole, Spoofing, Jamming, Tag cloning, Physical tampering
[14]	DDoS, Botnets, Falsified sensor data, Attacks on cloud services, Physical tampering
[15]	DDoS, Man-in-the-Middle, Spoofing, Physical tampering, Data breach, Malware, Ransomware
[16]	DDoS, Man-in-the-Middle, Malware, Ransomware, Physical tampering, Data breach, Spoofing
[17]	Physical damage, Exhaustion attacks, Cryptanalysis, Side-channel information, Man-in-the-Middle, DoS/DDoS, Message forging
[18]	Physical, Malware, DoS, Man-in-the-Middle, Replication, Spoofing, Injection, Social engineering
[19]	DoS, Man-in-the-Middle, Malware, Physical, Password
[20]	DoS, Man-in-the-Middle, Physical, Malware, Botnet, Spoofing, Eavesdropping
[21]	DDoS, Ransomware, Industrial spying, Click fraud

## Data Availability

Not applicable.
